# The influence of genetic architecture on responses to selection under drought in rice

**DOI:** 10.1111/eva.13419

**Published:** 2022-06-06

**Authors:** Irina Ćalić, Simon C. Groen, Jae Young Choi, Zoé Joly‐Lopez, Elena Hamann, Mignon A. Natividad, Katherine Dorph, Carlo Leo U. Cabral, Rolando O. Torres, Georgina V. Vergara, Amelia Henry, Michael D. Purugganan, Steven J. Franks

**Affiliations:** ^1^ Department of Biological Sciences Fordham University Bronx New York USA; ^2^ Institute of Botany University of Cologne Cologne Germany; ^3^ Department of Nematology University of California at Riverside Riverside California USA; ^4^ Department of Biology, Center for Genomics and Systems Biology New York University New York New York USA; ^5^ Département de Chimie Université du Québec à Montréal Québec Canada; ^6^ Department of Genetics and Odum School of Ecology University of Georgia Athens Georgia USA; ^7^ International Rice Research Institute Los Baños Laguna Philippines; ^8^ Institute of Crop Science University of the Philippines Los Baños Los Baños Laguna Philippines; ^9^ Center for Genomics and Systems Biology NYU Abu Dhabi Research Institute, New York University Abu Dhabi Abu Dhabi United Arab Emirates

**Keywords:** drought resistance, genetic architecture, G‐matrix, natural selection, *Oryza sativa*, pleiotropy

## Abstract

Accurately predicting responses to selection is a major goal in biology and important for successful crop breeding in changing environments. However, evolutionary responses to selection can be constrained by such factors as genetic and cross‐environment correlations, linkage, and pleiotropy, and our understanding of the extent and impact of such constraints is still developing. Here, we conducted a field experiment to investigate potential constraints to selection for drought resistance in rice (*Oryza sativa*) using phenotypic selection analysis and quantitative genetics. We found that traits related to drought response were heritable, and some were under selection, including selection for earlier flowering, which could allow drought escape. However, patterns of selection generally were not opposite under wet and dry conditions, and we did not find individual or closely linked genes that influenced multiple traits, indicating a lack of evidence that antagonistic pleiotropy, linkage, or cross‐environment correlations would constrain selection for drought resistance. In most cases, genetic correlations had little influence on responses to selection, with direct and indirect selection largely congruent. The exception to this was seed mass under drought, which was predicted to evolve in the opposite direction of direct selection due to correlations. Because of this indirect effect on selection on seed mass, selection for drought resistance was not accompanied by a decrease in seed mass, and yield increased with fecundity. Furthermore, breeding lines with high fitness and yield under drought also had high fitness and yield under wet conditions, indicating that there was no evidence for a yield penalty on drought resistance. We found multiple genes in which expression influenced both water use efficiency (WUE) and days to first flowering, supporting a genetic basis for the trade‐off between drought escape and avoidance strategies. Together, these results can provide helpful guidance for understanding and managing evolutionary constraints and breeding stress‐resistant crops.

## 1 INTRODUCTION

Understanding how traits evolve in response to selection under changing environmental conditions is a central goal of evolutionary biology. It is also crucial for predicting responses to climatic changes in natural and agricultural systems and for improving crop yields in increasingly variable environments (Challinor et al., 2014; Ensing et al., 2021; Gauzere et al., 2020). There are a limited number of cases (Franks et al., 2014) that have documented evolutionary responses to climatic changes such as shifts to earlier flowering time in response to increased temperatures and decreased precipitation (Anderson et al., 2012; Ehrlén & Valdés, 2020; Franks et al., 2007). While such evolutionary responses to climate change can potentially be very rapid, particularly when selection is strong, there can also be constraints that limit evolutionary responses (Etterson & Shaw, 2001). However, despite a relevant theoretical framework from quantitative genetics and increases in the number of species with well‐annotated reference genomes and genomic information from individuals, we still have a limited understanding of the degree to which genetic architecture constrains evolutionary responses to selection under varying conditions (Hansen, 2006). Improving this understanding is crucial for predicting evolutionary responses to climate change and for breeding crops that can tolerate conditions caused by climatic stresses (Ceccarelli & Grando, 2020; Snowdon et al., 2021).

The quantitative genetics approach to estimating evolutionary responses to selection is to apply the multivariate Breeder's equation, Δ*z* = *Gβ*, where Δ*z* is the evolutionary change in a set of traits, *G* is the matrix of additive genetic variances (heritabilities) and covariances (co‐heritabilities) among the traits, and *β* is the vector of selection gradients for a set of traits that give the covariances between the traits and fitness (Conner & Hartl, 2004; Lande & Arnold, 1983). This equation shows that the response to selection is proportional to the heritability of a trait and the strength of selection upon it. In addition, it is clear from this approach that evolution can occur if the direction of selection dramatically changes over space or time, as when drought on the Galapagos Islands changed patterns of selection, leading to rapid evolution of beak morphology in Darwin's finches (Grant & Grant, 1993, 2014). Similarly, variation in patterns of selection over space can potentially result in phenotypic divergence among populations and underlie local adaptation (Kawecki & Ebert, 2004). Furthermore, the multivariate Breeder's equation illustrates how constraints to evolution can occur when genetic covariances, given in *G*, oppose selection, given in *β* (Conner, 2012). When genetic architecture constrains selection, there is less of an evolutionary response to selection than if traits were uncorrelated. For example, genetic correlations were predicted to constrain evolutionary responses to selection by changes in climatic conditions in the annual plant *Chamaecrista fasciculata* (Etterson & Shaw, 2001).

If evolutionary responses are constrained by genetic correlations that oppose selection, there are several possible reasons for this. The genetic correlations could be caused by the same genes acting pleiotropically, genes that are physically linked, or genes that are physically unlinked but statistically associated. Although these possibilities are not mutually exclusive (Saltz et al., 2017), a deeper understanding of evolutionary constraints involves determining the genetic architecture of the traits (Falconer & MacKay, 1996; Lynch & Walsh, 1998). Another type of constraint can occur when selection favors alleles that lead to certain trait values under one set of conditions but opposes those trait values under a different set of conditions, which is known as antagonistic pleiotropy (Kawecki & Ebert, 2004). For example, if there is an allele that increases plant height, and greater plant height is favored under high‐density conditions but disfavored under low‐density conditions, this would be antagonistic pleiotropy. Alternatively, if a trait value is favored in one environment but unrelated to fitness in another environment, this is known as conditional neutrality (Anderson et al., 2013). Antagonistic pleiotropy can constrain evolutionary responses because the optimal phenotype under one set of conditions is suboptimal under other conditions, making it more difficult for selection to maximize fitness across contrasting environments over space, or when conditions fluctuate over time (Kawecki & Ebert, 2004).

Information on genetic architecture can provide insights into genetic constraints caused by genetic correlations or antagonistic pleiotropy and is particularly useful in plant and animal breeding (Kelly, 2009). Specifically, such information would help breeders determine whether selection on a set of genes, or selection under one set of conditions, would result in a yield penalty given potential correlated responses of other genes. However, quantitative genetic analysis is rarely combined with genomics to obtain a detailed picture of the influence of genetic architecture on evolutionary constraints (Dutta et al., 2021; Kelly, 2009; Yang et al., 2019).

Rice (*Oryza sativa*) provides a useful model system to study constraints on selection and how these may differ in environments that vary in water availability. Traditional varieties or landraces from the Indica and Japonica varietal groups have evolved for thousands of years in the fields of smallholder farmers, where they either received relatively consistent supplies of water under irrigation or experienced intermittent drought under rainfed conditions (Gutaker et al., 2020; Wing et al., 2018). Moreover, even before more recent expansions into Africa, Europe, and the Americas, rice was already grown along gradients of water availability across much of South, Southeast, and East Asia, so it is no surprise that the species harbors considerable phenotypic variation for traits that may influence drought resistance, including tillering (the production of stems that may bear panicles and flowers), water use efficiency, leaf rolling, accumulation of nonstructural carbohydrates, and flowering time (Cal et al., 2019; Groen et al., 2020; Kumar et al., 2014; Rebolledo et al., 2013, 2015; Robin et al., 2003; Torres et al., 2013; Xia et al., 2019).

We conducted a large‐scale field experiment in which 132 Indica and 84 Japonica accessions (the majority of which were inbred landraces supplemented with a smaller set of modern varieties or breeding lines) were subjected to dry or wet conditions. In a previous study from this experiment, we measured fitness and gene expression throughout the genome and found not only that heritability of gene expression was high, but also that although selection was neutral for the majority of transcripts, expression level was under selection for some genes, including genes likely involved in stress response (Groen et al., 2020). Furthermore, the prior study found more evidence for conditional neutrality than for antagonistic pleiotropy with respect to the direction of selection on gene expression in different environments (Groen et al., 2020). Building on this prior work, the current study focused on selection on functional traits, rather than gene expression, and investigated whether there were constraints to selection such as caused by genetic correlations or antagonistic pleiotropy. This work informs a basic understanding of constraints on responses to selection and can aid in agriculture by providing insights into how yields can be improved and maintained across different or changing environments.

## 2 METHODS

### 2.1 Field experiment

The field experiment was described previously (Groen et al., 2020). Briefly, we selected 132 Indica and 84 Japonica accessions as entries in the experiment, which predominantly were traditional varieties or landraces, whereas a smaller subset consisted of 18 modern varieties or breeding lines (Table S1). Among the breeding lines were two accessions that served as known drought‐susceptible and drought‐resistant “checks.” These were Indica accessions IR64 and Sahod Ulan 1, respectively, and were additionally replicated twice to bring the number of Indica entries to 136. Our experimental design was an alpha lattice design, which is an incomplete block design with cyclical permutations of treatments, with 22 blocks × 10 plots/block × three replicates, providing space for planting 220 entries in triplicate across a total of 660 plots per field environment. For each plot, a single entry of *O. sativa* was randomly assigned to generate the experiment design, and per plot 10 plants were placed individually within a row of 10 hills spaced 0.2 m × 0.2 m apart. The first and last plants of the same row served as border plants and were not used for phenotyping.

The field experiment took place in the 2016 dry season at the International Rice Research Institute (IRRI) in Los Baños, Laguna, Philippines. At 17 days after sowing (DAS) onto a seedbed, seedlings were pulled and transplanted into two flooded paddy fields. The first field remained flooded as a wet environment, while the second, located in a rainout shelter, was drained at 33 DAS to start the drought stress treatment. This dry environment was flash re‐flooded at 53, 64, and 91 DAS to give plants intermittent breaks from drought during the rest of the season. The drought stress treatment was designed to mimic occurrences of vegetative‐ and reproductive‐stage drought in rice fields of the rainfed lowland agro‐ecosystem across South and Southeast Asia (Kumar et al., 2014). Leaf tissue was collected at 50 DAS on one plant each for the first replicate plot of all 220 entries for DNA sequencing (Groen et al., 2020).

### 2.2 Trait measurements

A set of developmental, physiological, and life history traits were measured to assess individual and genotypic differences in drought response. We counted tiller number (TNR) for plants from the second hill in each plot at 53 and 56 DAS in the wet and dry environments, respectively. A separate sample of 10 young, fully expanded leaves was taken from the second hill in each plot on 51, 52, and 53 DAS (replicates 1, 2, and 3) in the dry environment, and tenth hill in each plot on 66 DAS in the wet environment, so that carbon isotope discrimination measurements could be performed as a proxy for water use efficiency (WUE). Leaf samples for carbon isotope analysis were dried, ground, and submitted to IRRI's Analytical Service Laboratory where they were analyzed using gas chromatography and isotope ratio mass spectrometry (GC‐IRMS). Analysis of Δ^13^C was determined using a wheat flour standard calibrated to Vienna Pee Dee Belemnite (Elemental Microanalysis).

We measured leaf osmotic potential (LOP) in both environments by collecting two fully expanded, young leaves from the first hill of each plot at 54 DAS between 10:00 h and 12:00 h. The samples were frozen at −10°C and then thawed at 25°C. Osmotic potential of 10 μl of expressed sap was measured using an osmometer (Vapro Osmometer, Wescor).

Xylem hydraulics (XHS) or sap exudation rate was measured in the dry environment at 51, 52, and 53 DAS for replicates 1, 2, and 3 from the second hill in each plot according to Morita and Abe (2002), and Henry et al. (2012). For this, stems of the entire plant were cut approximately 15 cm from the soil surface. A preweighed towel was wrapped around the cut stems and was covered and secured with a plastic bag and a rubber band. For plants with single tillers or a relatively small number of tillers, sap was collected by using a preweighed cotton‐filled 5‐ml centrifuge tube that was placed over the cut stems. The xylem sap exudation rate measurement was initiated at 07:20 h, and placement of the collection material was completed in 20 min on each day of measurement. Towels and cotton‐filled 5‐ml centrifuge tubes were collected after 4 h and re‐weighed to determine the amount of sap collected.

From the wet and dry environments, three tillers of the oven‐dried, harvested shoots from 4th‐hill samples were separated. Leaf blades were removed from each tiller, and approximately 8 cm of the stems were finely ground to be used for the determination of soluble sugar concentration (SSC) by Fourier Transform Infrared Spectroscopy (FTIR).

Leaf rolling and drying (LRO) were scored at 84 DAS in the dry environment by visual ratings according to the Standard Evaluation System for Rice (International Network for Genetic Evaluation of Rice, 1996). Measurements of days until flowering (DTF) were taken as described by Groen et al. (2020), where they were recorded as the day on which 50% of plants in a plot flowered. All in all, for each varietal group panel we measured a total of six traits in both fields and an additional two traits in the dry field.

### 2.3 Fitness component measurements

We measured two fitness components: flowering success and fecundity. Flowering success, as a binary trait, was 1 if a plant was able to produce at least one filled grain before the end of the growing season, and 0 if not (Anderson et al., 2013). Fecundity was assessed by sorting and counting filled, partially filled, and unfilled grains with the use of a seed counter (Hoffman Manufacturing), except for seeds with awns, which were counted manually. The data on grain numbers were published previously (Groen et al., 2020). The weights of filled grains were obtained after drying at 45°C for 3 days so that thousand‐grain weight (TGW) and yield (in grams for five hills per plot) could be calculated.

### 2.4 Phenotypic selection analyses

We measured the strength of selection on traits separately for the Indica and Japonica panels in each of the two field environments using regression‐based selection analysis (Lande, 1979; Lande & Arnold, 1983) as in Groen et al. (2020), except that here, we added block as a random factor in our selection analysis because we detected a significant block effect for WUE among the Indica panel populations (degrees of freedom = 21, *F* = 15.46, *p* < 0.001). We used phenotypic selection analysis, in which each individual was considered a replicate (Lande & Arnold, 1983). Fitness consisted of two multiplicative components (Conner, 1988): flowering success and fecundity. Flowering success was only measured in the dry field because these conditions caused some plants to fail to produce seeds. In the wet field, we did not analyze flowering success, but we removed individuals with zero fecundity fitness (no filled grains produced) from the analysis—59 Indica and 33 Japonica individuals were too few for selection analysis on flowering success and left fecundity fitness as a proxy for total lifetime fitness. We quantified fecundity in both wet and dry fields as the numbers of filled grains that individuals produced. For selection analyses using the fecundity fitness component, the filled grain number for each individual plant was normalized by dividing by the mean filled grain number of the population (a varietal group panel in a certain environment) after filtering out individuals with zero fecundity fitness in the previous step: *w'* = *w*_*i*_/mean(*w*). After this, the trait values across individuals were standardized by subtracting the population trait mean and dividing by the s.d. of the trait over the population: *z* = (*x*_*i*_ − mean[*x*])/SD (*x*) (Table S2; Lande, 1979).

We then conducted multivariate selection analyses (Lande & Arnold, 1983) for fecundity in the wet and dry environment using a custom script in *R* version 4.0 (R Core Team, 2016) (Supporting Information). We also conducted separate analyses to estimate the strength and direction of selection on all traits with the fitness component flowering success (a binary trait) using logistic regression for each trait across all individuals in the populations in the dry field environment (Janzen & Stern, 1998), again using a custom *R* script (Supporting Information). In both types of selection analyses, we calculated the linear selection gradients, (*β* = *P* ^−1^ *S*), as well as the quadratic selection gradients, (*γ* = *P* ^−1^ *C P* ^−1^), in which *P* represents the phenotypic variance–covariance matrix of traits (Lande & Arnold, 1983). *S* and *C* are the standardized directional and quadratic selection differentials *S* = Cov[*w*, *z*] and *C* = Cov[*w*,(*z* – mean(*z*)(*z* – mean(*z*))^*T*^)], respectively, which we had initially obtained from linear and logistic regressions in the mixed modeling package lme4 (Bates et al., 2015) in *R*. The mixed models incorporate both fixed and random effects, allowing us to evaluate the conditional mean of the fitness response while accounting for phenotypic correlations between traits (Bates et al., 2015). Estimates of *S* were obtained using the following univariate linear mixed model: *y* ~ *x* + *I*(1|*b*), where *y* is fitness; *x* is the trait measured and *b* is block (note that 1|signifies that factors are random in the “lme4” work package of the software *R*). Estimates of *C* were obtained using a quadratic mixed model with the same parameters: *y* ~ *x* + *I*(*x* ^2^) + *I*(1|*b*) (note that 1|signifies that factors are random in the “lme4” work package of the software *R*) (Table S6).

The selection differentials reflect the total (direct and indirect) strength of selection on the phenotypic values, while the selection gradients only reflect the former (Kingsolver et al., 2001; Lande & Arnold, 1983). To establish selection gradients and estimates of the standard error on these selection gradients for total lifetime fitness in dry conditions, we summed the selection gradients as well as the estimates of the standard error on these selection gradients for the fitness components “flowering success” and “fecundity” under drought conditions (Koenig et al., 1991). This was appropriate because in our study, flowering success and fecundity are multiplicative fitness components and flowering success is a binary trait (Conner, 1988; Koenig et al., 1991).

### 2.5 Collection of population genome re‐sequencing data for G‐matrix estimation

Raw FASTQ reads from 27 accessions included in the 3 K‐RG project and from 170 accessions included in our previous reconstruction of rice's dispersal history were downloaded from the Sequence Read Archive (SRA) website under BioProject ID numbers PRJEB6180, PRJNA422249, and PRJNA557122, respectively (Gutaker et al., 2020; Wang et al., 2018). The 3 K‐RG project refers to the 3000 rice genomes project, in which the genomes of a core collection of 3010 rice accessions from 89 countries were re‐sequenced to an average sequence depth of 14×. From sequencing these, a total of approximately 18.9 million single‐nucleotide polymorphisms (SNPs) were discovered when data were aligned to the reference genome of the temperate japonica accession Nipponbare (Wang et al., 2018).

DNA for genome re‐sequencing of the remaining 18 accessions in our panel was obtained from leaf tissue of plants grown at New York University in growth cabinets or at IRRI as part of this field experiment. To extract nucleic acids from these leaves, samples were ground using mortar and pestle in liquid nitrogen. DNA was extracted using the Qiagen DNeasy Plant Mini Kit following the manufacturer's protocol (QIAGEN, Hilden, Germany). Yields ranged between 3.5 and 56 ng μl^−1^. Extracted DNA from each sample was prepared for Illumina genome sequencing using the Illumina Nextera DNA Library Preparation Kit. Sequencing was done on the Illumina HiSeq 2500—HighOutput Mode v3 with 2 × 100 bp read configuration, at the New York University Genomics Core Facility. Raw FASTQ reads are available from SRA BioProject ID numbers PRJNA422249 and PRJNA557122.

Combined with the accessions included in the 3 K‐RG project, a total of 1,203,564,772,205 bps (~1.2 Tbps) were included in downstream analyses for a combined 215 accessions. The genomes of five of the 220 entries in the field experiment were not re‐sequenced: one entry was a “filler” accession, used to fill gaps in the experimental design and its genetic make‐up is not of interest, and a further four entries were replicated checks of two accessions, the known drought‐susceptible breeding line IR64 and the known drought‐resistant breeding line Sahod Ulan 1. Accession numbers and origins of tissue for DNA extraction can be found in Table S1.

### 2.6 Reference genome‐based DNA read alignment

FASTQ reads were preprocessed using BBTools (https://jgi.doe.gov/data‐and‐tools/bbtools/) bbduk program version 37.66 for read quality control and adapter trimming. For bbduk we used the option minlen = 25 qtrim = rl trimq = 10 ktrim = r k = 25 mink = 11 hdist = 1 tpe tbo, which trimmed reads below a phred score of 10 on both sides of the reads to a minimum length of 25 bps, trimmed 3′ adapters using a kmer size of 25 and using a kmer size of 11 for read ends, allowing one hamming distance mismatch, trimmed adapters based on overlapping regions of the paired end reads, and trimmed reads to equal lengths if one of them was adapter trimmed.

FASTQ reads were aligned to the reference *O. sativa* Nipponbare genome (temperate japonica) downloaded from EnsemblPlants release 36 (ftp://ftp.ensemblgenomes.org/pub/plants/). Read alignment was done using the program bwa‐mem version 0.7.16a‐r1181 (Li, 2013). Only the 12 pseudomolecules were used as a reference, and the unassembled scaffolds were left out. PCR duplicates during the library preparation step were determined computationally and removed using the program picard version 2.9.0 (http://broadinstitute.github.io/picard/).

### 2.7 SNP calling

For each accession, genotype calling for each site was conducted using the GATK HaplotypeCaller engine in the ‘‐ERC GVCF’ mode and genotypes were output in the genomic variant call format (gVCF). The gVCFs from each sample were merged to conduct a multi‐sample joint genotyping using the GATK GenotypeGVCFs engine. Genotypes were divided into SNP or insertion/deletion (INDEL) variants and filtered using the GATK best‐practice hard filter pipeline (Van der Auwera et al., 2013). For SNP variants, we excluded regions that overlapped repetitive regions and variants that were within 5 bps of an INDEL variant. We then used vcftools version 0.1.15 to select SNPs that had at least 80% of the sites with a genotype call and exclude SNPs with minor allele frequency (MAF) <5% to remove potential false‐positive SNP calls arising from sequencing errors or false genotype calls (Danecek et al., 2011).

Before conducting downstream analyses based on the SNP data, we confirmed that the frequency of heterozygous loci for both the accessions and markers included in our association panels was low enough to assure a high level of inbreeding for each accession by running a series of QC filtering steps so that a high‐quality SNP set remained for each panel. Starting with a full pseudomolecule SNP set of 3,616,806 SNPs across 215 accessions, we applied further filters to retain SNPs with a read depth coverage of 1500‐4000, a call rate > 90%, and observed heterozygosity across loci of <10%, resulting in 1,217,446 SNPs in the remaining test SNP set. Accessions with an inbreeding coefficient of <70% homozygosity were removed for association mapping analyses, leaving 211 out of the 215 accessions (Figure S1a). We then continued filtering the SNP datasets for the Indica and Japonica varietal groups independently. We accounted for missing data through imputation and removed loci with MAF <5% and that were biallelic. This left final SNP sets consisting of 424,105 SNP loci across 128 Indica accessions and 377,819 loci across 83 Japonica accessions that remained for downstream analyses (Supporting Information). In addition, we performed principal component analysis (PCA) using genotype likelihoods to generate PCs that we would use to control for population structure in these downstream analyses (Supporting Information). The genotype posterior probabilities were obtained from an ANGSD command (Supporting Information). The genotype posterior probability was then used by the program ngsCovar to conduct the PCA (Fumagalli et al., 2014). As expected, PCA revealed two related, but distinct, subpopulation clusters for each varietal group panel: in Indica 103 clustered accessions belonged to the Indica subpopulation and 25 to the *circum*‐aus subpopulation (Figure S1b), whereas in Japonica 64 clustered accessions belonged to the Japonica subpopulation and 19 to the *circum*‐basmati subpopulation (Figure S1c). Since each subpopulation can be divided further into smaller groups along subsequent PC axes, we used four PCs per population in our GWAS to control for population structure (see Section 2.9).

### 2.8 G‐matrix estimation and prediction of the outcome of selection

Prior to estimating G‐matrices and other analyses that required genotypic means of traits, we calculated least‐square means (LS means) for each accession‐by‐environment combination, also known as population marginal means or adjusted means (Lenth, 2016; Searle et al., 1980), by using a custom *R* script (Table S2).

The G‐matrix gives the additive genetic variances and covariances for a set of traits. Estimates of additive genetic variance and covariance for each varietal group panel in each of the two environments were obtained following Tropf et al. (2015). First, we constructed kinship matrices from the separate SNP datasets for each varietal group panel using the VanRaden method in the *R* package GAPIT version 3, a genome association and prediction integrated tool (Lipka et al., 2012; VanRaden, 2008), after having pruned each SNP dataset by randomly choosing a single SNP per 1000 bps (Supporting Information). We let GAPIT estimate the contribution of structure between accessions within each varietal group panel to each trait measured in the wet and dry environment populations separately using a variance component model, providing us with the fraction of phenotypic variance explained by the kinship matrix in each environment. This fraction (termed pseudo‐heritability) resembles the narrow‐sense heritability (*h* ^*2*^) estimated from a pedigree and serves as an estimate of the additive genetic variance of a trait (Kang et al., 2010). We then applied a bivariate genetic model as previously outlined to obtain estimates of the additive genetic covariance between traits (Tropf et al., 2015).

We used the G‐matrix to predict the outcome of selection on trait values across one generation (Δz) by multiplying the matrix with the vector of linear selection gradients on the set of traits we measured as defined in the multivariate Breeder's equation: Δz = G *β*. We assessed whether evolutionary constraints were present for selection on each trait by comparing the predicted outcome of direct selection on the trait (given by multiplying *β* with the trait's genetic variance) with the predicted outcome of indirect selection on the trait (given by multiplying *β* with the sum of the trait's genetic covariances between the focal trait and other traits) for traits in which direct and indirect selection is in opposite directions.

### 2.9 Genome‐wide association study (GWAS)

For each phenotypic dataset from the wet and dry field environment populations, we conducted GWAS independently for both the Indica and Japonica varietal group panels. Multiple Locus Mixed Linear Model (MLMM) analysis (Segura et al., 2012) was conducted on the non‐LD‐pruned SNP datasets using GAPIT (Lipka et al., 2012). This model uses a forward–backward stepwise linear mixed‐model regression to include associated markers as covariates. To control for the effects of population structure on association mapping, we included as covariates in the model the top four principal components from the SNP dataset of the varietal group under study as well as the kinship matrix for that group described in Section 2.7 as a random factor. Quantile–quantile plots were automatically generated in GAPIT using the package qqman (Turner, 2018). Manhattan plots were produced in *R* using the Cmplot function (https://github.com/YinLiLin/R‐CMplot), and complete results have been deposited in Zenodo (DOI: 10.5281/zenodo.5513036).

Bonferroni‐corrected *p* values were set as significance thresholds (0.05/the total number of loci in each association test), which was 0.05/424,105 = 1.179 × 10^−7^ in Indica and 0.05/377,819 = 1.323 × 10^−7^ in Japonica. In addition, we considered more lenient thresholds, which we computed following the SimpleM multiple testing correction method (Gao et al., 2008; Gao et al., 2010) using a custom *R* script (0.05/the number of independent tests; Supporting Information). For Indica, the less stringent SimpleM significance threshold was based on an inferred number of effective loci (Inferred Meff) of 38,822, leading to a *p* value threshold of 0.05/38,822 = 1.288 × 10^−6^. For Japonica this threshold was based on an Inferred Meff of 24,765, leading to a *p* value threshold of 0.05/24,765 = 2.019 × 10^−6^.

To gain insight in the functional roles of potential gene candidates in regulating drought stress responses, we searched the Rice Annotation Project Database (https://rapdb.dna.affrc.go.jp). We considered genes as candidates when their physical position was within a genomic region of 50 kbp upstream and 50 kbp downstream around an association peak. This window size was chosen conservatively given an estimated breakdown of linkage disequilibrium in a range of 75–125 kbp in *O. sativa* subpopulation Indica, and even longer ranges in other *O. sativa* subpopulations (Huang et al., 2010; Mather et al., 2007; McNally et al., 2009; Zhao et al., 2011). Candidate genes were annotated using the *O. sativa* Nipponbare reference genome as background.

### 2.10 Transcript–trait association analysis

We identified leaf transcripts significantly associated in their expression with the traits we measured at the vegetative stage (all traits except TGW) for the Indica and Japonica populations separately in each field environment by using regression models: *Y* = *μ* + *T* + *ε*, in which *Y* represents the functional trait of interest, *μ* an intercept parameter, *T* the transcript covariate, and *ε* residual error. The transcript level data for 15,635 transcripts were obtained as part of this field experiment and published previously (Groen et al., 2020). Transcript abundances were measured from leaf blades of the same individual plants for which flowering success was assessed and filled grain numbers were counted, and this leaf tissue was harvested at the same time‐point at which LOP was measured in both field environments (54 DAS). Associations were deemed significant when they came within an order of magnitude of a Bonferroni threshold based on a transcript number of *n* = 15,635. For sets of transcripts associated with one or more traits, we performed gene set enrichment analysis, focusing on Gene Ontology (GO) biological processes, using PANTHER's Overrepresentation Test (released 2021‐02‐24) with the *O. sativa* genes in the GO database (DOI: 10.5281/zenodo.4495804; released 2021‐02‐01) as background gene set used to match the foreground set (Mi et al., 2021). Enrichment was calculated using Fisher's exact tests followed by FDR correction.

### 2.11 Genomic prediction

Genomic breeding values of Indica population accessions for total fitness in wet and dry conditions, data described by Groen et al. (2020), were determined by letting GAPIT estimate the best linear unbiased predictions (BLUPs) and associated prediction error variances (PEVs) for these fitness traits using methodology developed by Zhang et al. (2010), as described by Lipka et al. (2012).

## 3 RESULTS

### 3.1 Patterns of selection in dry and wet conditions

**FIGURE 1:**
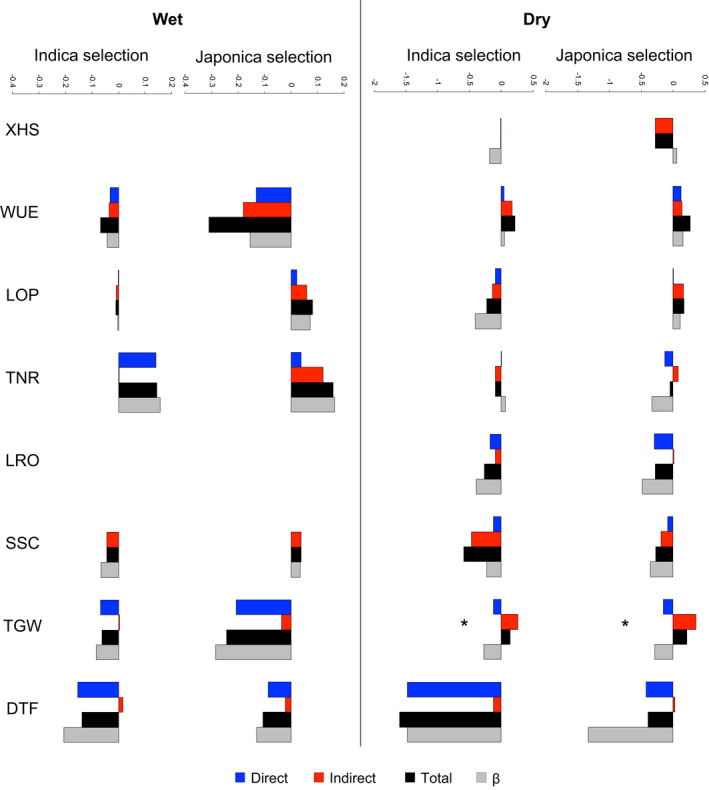
Predicted outcome of selection can differ in direction from the selection gradient *ß*. Each bar depicts the magnitude and direction of *ß* (gray; calculated as the covariance between trait and fitness values), compared with the predicted effects of direct selection (blue; calculated from the genetic variance of a trait and *ß*) and indirect selection (red; calculated from the genetic covariances of a trait with other traits and *ß*), and the total predicted outcome of selection (black; calculated from the sum of predicted direct and indirect effects). XHS, xylem hydraulics; WUE, water use efficiency; LOP, leaf osmotic potential; TNR, vegetative‐stage tiller number; LRO, leaf rolling; SSC, soluble sugar concentration; TGW, 1000‐grain weight; DTF, days until flowering. The asterisk denotes that the predicted effects of direct and indirect selection were in opposite direction and that the value of the predicted effect of indirect selection was larger than the error estimate on the selection gradient

Both the Indica and Japonica varietal group‐panel populations experienced similar selection under drought. In drought conditions, there was selection for earlier flowering, with the selection gradient (*ß*) on DTF −1.476 (±0.293 standard error [SE]) in Indica and −1.335 (±0.349) in Japonica (the negative selection gradient indicates selection for earlier flowering time), but no selection on drought avoidance‐related traits (Figure 1; Tables 1 and 2).

**TABLE 1:** Selection gradient (*β*), genetic variances and covariances (in the G‐matrix) and predicted outcome of selection (Δ*z*) for rice traits in experimental populations of the Indica varietal group grown in wet (top) and dry (bottom) conditions

Traits	Selection gradient		G‐matrix								Δ*z*		
	*β* (±SE)	*p* value	XHS	WUE	LOP	TNR	LRO	SSC	TGW	DTF	Direct	Indirect	Total
Wet													
WUE	**−0.042** (±0.033)	0.2003	NA	0.746	−0.016	**−0.182**	NA	0.002	0.169	−0.036	−0.032	−0.036	−0.067
LOP	**−0.002** (±0.033)	0.9428	NA	−0.016	0.117	−0.025	NA	0.065	−0.052	0.029	0.000	−0.009	−0.009
TNR	0.157 (±0.034)	0.0000	NA	**−0.182**	−0.025	0.897	NA	0.036	**−0.207**	0.096	0.141	0.003	0.144
SSC	−0.067 (±0.034)	0.0497	NA	0.002	0.065	0.036	NA	0.000	−0.051	**0.260**	0.000	−0.044	−0.044
TGW	−0.085 (±0.035)	0.0152	NA	0.169	−0.052	**−0.207**	NA	−0.051	0.799	**−0.198**	−0.068	0.005	−0.063
DTF	−0.206 (±0.036)	0.0000	NA	−0.036	0.029	0.096	NA	**0.260**	**−0.198**	0.751	−0.155	0.016	−0.139
Dry													
XHS	−0.181 (±0.259)	0.4855	0.000	−0.074	0.000	0.074	−0.063	0.064	0.057	0.001	0.000	−0.005	−0.005
WUE	0.054 (±0.259)	0.8357		0.833	−0.003	−0.119	0.019	−0.071	0.084	−0.120	0.045	0.170	0.215
LOP	−0.405 (±0.268)	0.1331			0.221	0.057	−0.017	0.063	−0.111	0.111	−0.089	−0.137	−0.226
TNR	0.065 (±0.272)	0.8107				0.078	0.078	0.031	0.088	−0.007	0.005	−0.095	−0.089
LRO	−0.392 (±0.259)	0.1324					0.439	−0.016	−0.126	0.107	−0.172	−0.095	−0.267
SSC	−0.228 (±0.259)	0.3803						0.537	0.088	**0.280**	−0.122	−0.469	−0.592
TGW	−0.271 (±0.250)	0.2780							0.443	−0.126	−0.120	0.260	0.139
DTF	−1.476 (±0.293)	0.0000								1.000	−1.476	−0.123	−1.599

*Note*: In the G‐matrix genetic variances (SNP‐based or narrow‐sense heritabilities) are on the diagonal, and genetic covariances on the off‐diagonal cells (significant covariances are indicated in bold; *p* < 0.05). Traits with significant *β* (*p* < 0.05) in one condition are conditionally neutral, except for DTF, which was nonantagonistically pleiotropic. None of the traits displayed antagonistic pleiotropy.

Abbreviations: DTF, Days until flowering; LOP, Leaf osmotic potential; LRO, Leaf rolling; SSC, Stem sugar content; TGW, 1000‐grain weight; TNR, Tiller number; WUE, Water use efficiency; XHS, Xylem hydraulics.

**TABLE 2:** Selection gradient (*β*), genetic variances and covariances (in the G‐matrix) and predicted outcome of selection (Δz) for rice traits in experimental populations of the Japonica varietal group grown in wet (top) and dry (bottom) conditions

Traits	Selection gradient		G‐matrix								Δz		
	*β* (±SE)	*p* value	XHS	WUE	LOP	TNR	LRO	SSC	TGW	DTF	Direct	Indirect	Total
Wet													
WUE	−0.155 (±0.076)	0.0433	NA	0.844	−0.176	**−0.438**	NA	−0.126	0.211	**0.241**	−0.131	−0.181	−0.311
LOP	0.072 (±0.069)	0.3037	NA	−0.176	0.316	0.079	NA	0.020	−0.039	−0.056	0.023	0.059	0.082
TNR	0.164 (±0.071)	0.0213	NA	**−0.438**	0.079	0.235	NA	0.069	−0.092	−0.146	0.039	0.121	0.160
SSC	**0.034** (±0.069)	0.6229	NA	−0.126	0.020	0.069	NA	0.000	−0.087	0.139	0.000	0.039	0.039
TGW	−0.285 (±0.071)	0.0001	NA	0.211	−0.039	−0.092	NA	−0.087	0.732	−0.140	−0.208	−0.035	−0.244
DTF	−0.130 (±0.071)	0.0671	NA	**0.241**	−0.056	−0.146	NA	0.139	−0.140	0.664	−0.086	−0.021	−0.107
Dry													
XHS	0.058 (±0.337)	0.8656	0	−0.069	−0.039	−0.056	0.006	−0.022	−0.037	**0.222**	0.000	−0.276	−0.276
WUE	0.154 (±0.344)	0.6575		0.830	−0.020	−0.185	−0.060	−0.020	0.103	−0.062	0.128	0.145	0.273
LOP	0.113 (±0.340)	0.7434			0.088	0.002	0.026	0.016	−0.134	−0.111	0.010	0.162	0.172
TNR	−0.334 (±0.290)	0.2583				0.381	0.177	0.038	−0.158	−0.124	−0.127	0.080	−0.047
LRO	−0.484 (±0.286)	0.1023					0.603	−0.018	−0.201	−0.008	−0.292	0.010	−0.282
SSC	−0.358 (±0.298)	0.2405						0.238	−0.039	0.144	−0.085	−0.187	−0.272
TGW	−0.290 (±0.319)	0.3712							0.509	−0.149	−0.148	0.362	0.215
DTF	−1.335 (±0.349)	0.0007								0.317	−0.423	0.028	−0.395

*Note*: In the G‐matrix genetic variances (SNP‐based or narrow‐sense heritabilities) are on the diagonal, and genetic covariances on the off‐diagonal cells (significant covariances are indicated in bold; *p* < 0.05). Traits with significant *β* in one condition (*p* < 0.05) are conditionally neutral. None of the traits displayed antagonistic pleiotropy.

Abbreviations: DTF, Days until flowering; LOP, Leaf osmotic potential; LRO, Leaf rolling; SSC, Stem sugar content; TGW, 1000‐grain weight; TNR, Tiller number; WUE, Water use efficiency; XHS, Xylem hydraulics.

In wet conditions, we detected significant linear selection in one or both varietal group‐panel populations on all traits except LOP (Figure 1; Tables 1 and 2). Both groups showed selection for smaller seed size with *ß* for TGW −0.085 (±0.035) in Indica and −0.285 (±0.071) in Japonica. There was also selection for increased tiller number in both groups, with *ß* for TNR 0.157 (±0.034) in Indica and 0.164 (±0.071) in Japonica (Figure 1; Tables 1 and 2). WUE was under negative selection in Japonica (*ß* = −0.155 [±0.076]) and SSC was under negative selection in Indica (*ß* = −0.067 [±0.034]) (Figure 1; Tables 1 and 2). The direction of selection was the same in both varietal groups for all traits.

Overall, patterns of selection on functional traits were similar in both environments (Figure 1; Tables 1 and 2). Of the traits experiencing selection, only DTF showed significant values for *ß* in both the wet and dry environment, but these were in the same direction (selection for earlier flowering). The selection gradients on DTF were substantially lower in magnitude in the wet compared with dry conditions, indicating that selection for earlier flowering was stronger under dry compared with wet conditions (Tables 1 and 2). There were no cases in which the direction of selection was opposite in the different environments (Tables 1 and 2).

### 3.2 Genetic architecture and responses to selection

We found that in the panels of both varietal groups, almost all traits were heritable for the populations in each environment, except for SSC in wet conditions and XHS (only measured under drought) in dry conditions (Tables 1 and 2). There were also significant additive genetic covariances among many traits, with the G‐matrices for the populations of each varietal group panel in each environment given in Tables 1 and 2.

An analysis of the selection gradients showed both direct and indirect selection for nearly all traits (Figure 1). Indirect selection is influenced by genetic correlations, but direct selection is not, so a comparison of direct and indirect selection indicates the effects of genetic correlations on the predicted outcome of selection. For most traits, indirect selection was relatively weak and often consistent with the direction of direct selection (Figure 1). However, there was one major exception. In both the Indica and Japonica panel populations under drought, seed mass (TGW) showed negative direct selection but positive indirect selection, which was strong enough that the predicted response to selection was positive rather than negative (Figure 1).

### 3.3 Pleiotropy and linkage

**FIGURE 2:**
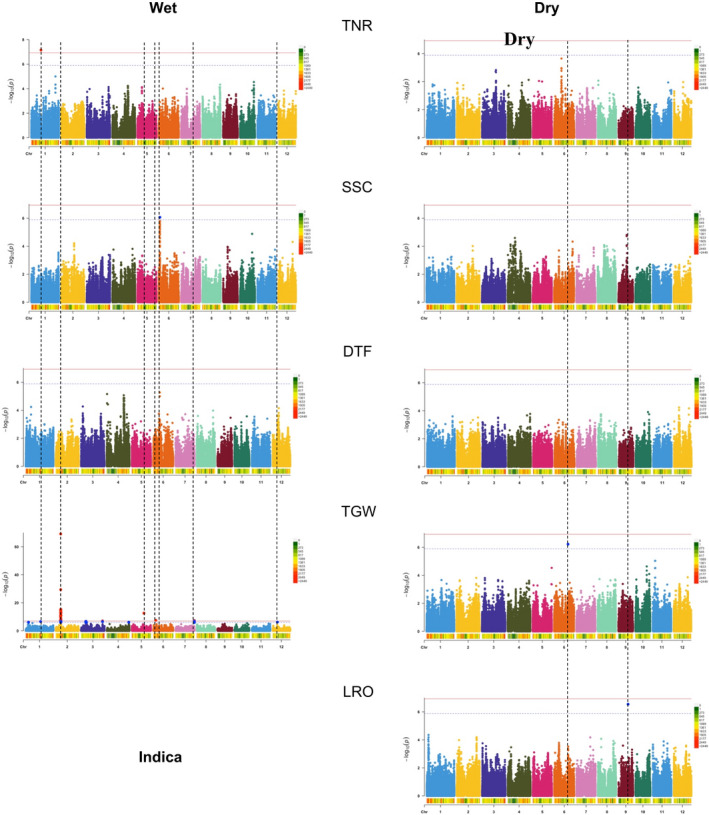
Multi‐trait GWAS for experimental populations of the Indica varietal group grown in wet and dry conditions. SNPs in peaks whose *p* value—depicted as –log_10_(*P*) on the y axis—passed the Bonferroni threshold (red horizontal line) or the less stringent SimpleM threshold (blue horizontal line) are marked with black vertical lines to facilitate cross‐trait comparisons of SNP associations. The colored bar underneath the *x* axis of each Manhattan plot shows the SNP density per every 1 Mbp. TNR, vegetative‐stage tiller number; SSC, soluble sugar concentration; DTF, days until flowering; TGW, 1000‐grain weight; LRO, leaf rolling

GWAS detected some associations between genes and traits, but the results differed among varietal group panels, environments, and the significance threshold used. For the Indica panel population in the wet environment, there were significant SNP associations (exceeding the Bonferroni threshold) for TNR and TGW, with one significant association for each of these two traits (Figure 2). None of the other traits showed SNP associations that breached the Bonferroni threshold in this environment (Figures S2 and S3). However, when we used a more lenient SimpleM threshold, SNP associations became apparent for SSC (Figure 2). For the Indica panel population in the dry environment, no trait exhibited Bonferroni threshold‐surpassing *p* values (Figures 2, S2 and S3), but TGW and LRO had SNP associations that passed the SimpleM threshold (Figure 2).

**FIGURE 3:**
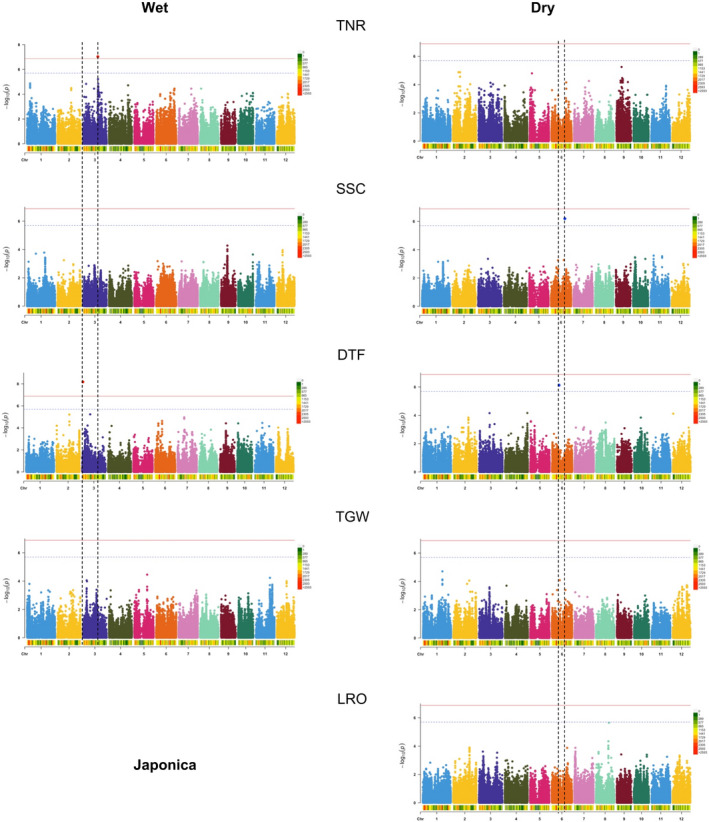
Multi‐trait GWAS for experimental populations of the Japonica varietal group grown in wet and dry conditions. SNPs in peaks whose *p* value—depicted as –log_10_(*P*) on the *y* axis—passed the Bonferroni threshold (red horizontal line) or the less stringent SimpleM threshold (blue horizontal line) are marked with black vertical lines to facilitate cross‐trait comparisons of SNP associations. The colored bar underneath the x axis of each Manhattan plot shows the SNP density per every 1 Mbp. TNR, vegetative‐stage tiller number; SSC, soluble sugar concentration; DTF, days until flowering; TGW, 1000‐grain weight; LRO, leaf rolling

For the Japonica panel population in the wet environment, there were significant (exceeded the Bonferroni threshold) SNP associations for TNR and DTF (Figures 3, S4 and S5). In the dry environment, we only observed associations for DTF and SSC when we used a more lenient SimpleM threshold (Figures 3, S4 and S5). Candidate genes around each SNP association can be found in Table S3 and descriptions of these candidate genes in the Supporting Information.

There were no cases in which a genetic locus was associated with more than one trait, for the populations of either varietal group panel in either environment, even when the more lenient SimpleM threshold was used.

### 3.4 Gene expression and genetic correlations

We found significant associations between expression levels of some genes and traits, which differed among populations of each varietal group panel across environments, with more significant associations observed in the dry environment (Table S4). For the population of the Indica panel in wet conditions, we detected a transcript association shared between two traits, SSC and DTF. The underlying gene is *DWARF18*/*OsGA3ox2* (Os01g0177400). In the population of the Japonica panel in wet conditions, expression levels of *Heading date3a* (Os06g0157700) were associated with DTF and LOP. The largest sets of overlapping transcripts were found for WUE and DTF (9 transcripts) (Figure S6).

**FIGURE 4:**
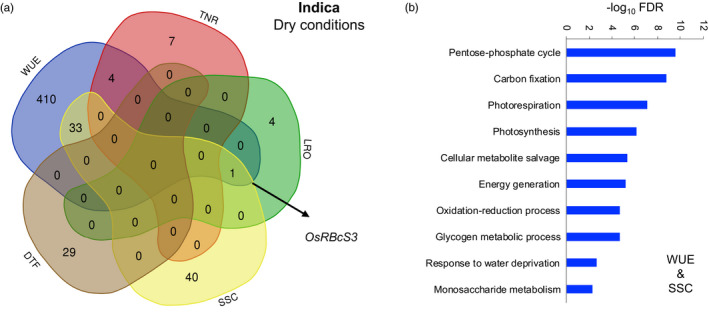
Multi‐trait analysis of transcript–trait associations for an experimental population of the Indica varietal group grown in water‐limited conditions. (a) The Venn diagram depicts the number of trait associations for transcript levels that are shared between traits. Only traits with significant transcript associations are depicted. (b) Gene ontology (GO) biological processes that are enriched among transcripts whose levels are associated with water use efficiency (WUE) and soluble sugar concentration (SSC). DTF, days until flowering; LRO, leaf rolling; TNR, vegetative‐stage tiller number

For the population of the Indica panel under drought, there were expression associations with WUE, TNR, LRO, SSC, and DTF. Most transcripts that showed significant associations were associated with only one trait. However, we identified one transcript that was linked to three traits: LRO, SSC, and WUE (Figure 4a). The underlying gene is Os12g0291200, which encodes a small subunit of RuBisCO and is involved in photosynthesis. For the populations of both the Indica and Japonica panels in the dry environment, there were substantial overlaps in significant associations between transcript expression levels and WUE as well as SSC (Figure 4a, Table S4). In both varietal groups, these shared transcripts were enriched for ones related to photosynthesis and responses to water deprivation (Figure 4b, Table S4). In the population of the Japonica panel under drought, TNR and WUE also showed a relatively high number of overlapping transcripts (13 in total), and one encodes OsSerpin (OS03T0610800–01). Further descriptions of transcript associations can be found in the Supporting Information.

### 3.5 Rice yield in wet and dry conditions

**FIGURE 5:**
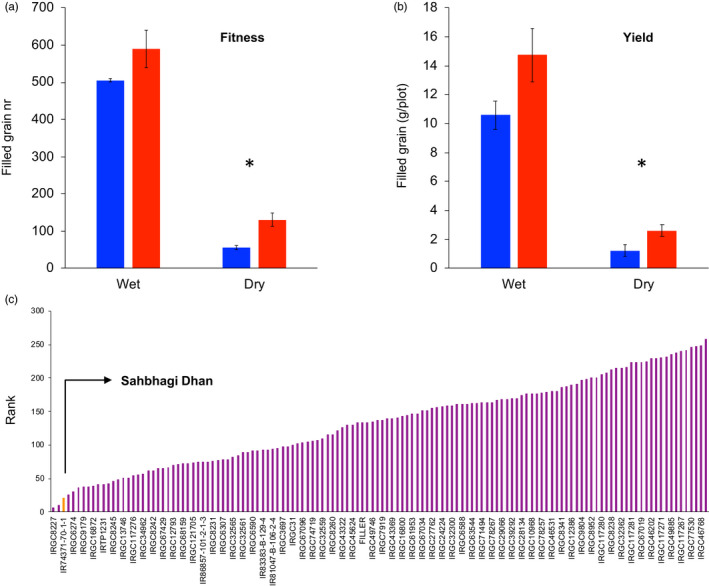
Breeding lines for rainfed environments are more drought resistant than ones for irrigated environments and do not show fitness or yield penalties. (a) Fitness (filled grain number), and (b) yield are significantly higher (*p* < 0.05) among breeding lines targeted at rainfed environments (red) than among lines targeted at irrigated environments (blue), when grown in dry conditions, but not when grown in wet conditions. (c) Cumulative ranks of genomic prediction values for fitness in wet and dry field conditions. Accessions with the highest fitness across fields have the lowest ranks

To determine how selection for drought resistance may influence yield in both the wet and dry environments, we re‐sequenced the genomes and analyzed the breeding values (from the DNA profiles), fitness, and yield of 18 Indica breeding lines selected for irrigated and rainfed environments (Table 3). Breeding lines for drought resistance in rainfed environments showed a significant improvement, relative to lines bred for irrigated environments, in yield and fitness under drought (Figure 5a,b). In addition, these drought‐resistant lines did not show reduced fitness (Figure 5a) or yield (Figure 5b) relative to lines bred for irrigated environments under wet conditions. Notably, the breeding line that ranked top for showing the highest breeding value for fitness (Sahbhagi Dhan) not only did so under drought, but also in wet conditions (Figure 5c, Table S5).

**TABLE 3:** Genomes of 18 Indica breeding lines intended for agro‐ecosystems with different water availabilities were re‐sequenced and used to estimate breeding values for fitness in wet and dry field conditions using genomic prediction. The breeding lines were then ranked relative to landraces that evolved in fields of smallholder farmers according to their fitness with high‐fitness genotypes receiving the lowest scores, that is, these were ranked top. The line with the lowest cumulative rank was the most stable for high fitness in the wet and dry field environments

IRGC ID NR	Accession name	Ecosystem	Wet fitness rank	Dry fitness rank	Cumulative fitness rank
IR74371‐70‐1‐1	Sahbhagi Dhan	RL	13	8	21
IRGC 122451	Sahod Ulan 1	RL	37	19	56
IR82589‐B‐B‐84‐3	BRRI dhan71	RL	36	29	65
IR86857‐101‐2‐1‐3	Katihan 3 (NSIC Rc27)	UP	42	33	75
IR79913‐B‐176‐B‐4	Katihan 1	UP	49	26	75
IR83383‐B‐129‐4	Sukha Dhan 6	RL	30	63	93
IR81047‐B‐106‐2‐4	Sahod Ulan 12	RL	33	61	94
IR86781‐3‐3‐1‐1	Sahod Ulan 20	RL	44	54	98
IR81023‐B‐116‐1‐2	Sahod Ulan 5	RL	83	47	130
IR81412‐B‐B‐82–1	Sahod Ulan 3	RL	34	110	144
IR83380‐B‐B‐124‐3	sister of CR Dhan 201 (IR83380‐B‐B‐124‐1)	UP	125	39	164
IRTP 13772	PSBRC_1	UP	102	74	176
IRGC 9790	BPI 76	IR	43	104	147
IRTP 9542	IR58	IR	89	72	161
IRIS 66–333,787	FL478	IR	113	56	169
IRGC 39292	IR36	IR	75	94	169
IRGC 66970	IR64	IR	99	92	191
IRTP 201	IR29	IR	96	112	208

*Note*: BLUPs for fitness in wet and dry conditions were ranked for Indica breeding lines in relation to a further 114 Indica varietal group accessions.

Abbreviations: IR, Irrigated; RL, Rainfed Lowland; UP, Upland.

## 4 DISCUSSION

Understanding the mechanisms behind genetic and phenotypic change in populations over time is a major challenge in evolutionary biology and of significant practical importance in crop breeding.

In the present study, we performed phenotypic selection analysis and genomic association studies on traits associated with drought response with the aim of understanding patterns of selection and potential constraints to evolution of these traits in Indica and Japonica rice populations that evolved under different water availabilities in the fields of smallholder farmers across Asia.

### 4.1 Selection for drought escape

We found strong selection for earlier flowering under drought, which is consistent with the strategy of drought escape (Kooyers, 2015). A substantial number of other studies have found selection on or evolution of drought escape through earlier onset of flowering, particularly under late‐season drought conditions (Franks et al., 2007; Hamann et al., 2018; Ivey & Carr, 2012; Johnson et al., 2021; Lambrecht et al., 2020; Sherrard & Maherali, 2006). However, other studies have shown evolution of drought avoidance rather than escape or evolutionary shifts to later flowering following drought (Anstett et al., 2021) or a lack of evolutionary response to drought (Vtipil & Sheth, 2020). Which strategy is favored is likely to depend on the environmental conditions and pattern of drought, as well as on the characteristics of the organism (Burnette & Eckhart, 2021; Kooyers et al., 2021). Interestingly, earlier flowering was favored in our study despite the fact that drought was implemented mostly throughout the study, rather than in a pattern of decreasing moisture availability, although the plants may have experienced increasing negative effects of the drought over the course of the growing season, and did experience saturating conditions early in the experiment. Furthermore, earlier flowering was favored even under wet conditions, in which case this pattern of selection does not appear to be due to drought response but because earlier flowering was favored for other reasons. Aside from drought escape, selection for earlier flowering appears widespread, and earlier phenology can often be favored or appear to be favored for a number of reasons (Austen et al., 2017). Our results indicate that in terms of breeding rice and other crops, selection for earlier flowering may enhance yield under drought conditions and could potentially be favored under wetter conditions as well, but this would depend on the specifics of the system.

### 4.2 Response to selection was generally not constrained by genetic correlations

When genetic correlations oppose the direction of selection, evolutionary responses can be constrained (Conner, 2012; Etterson & Shaw, 2001; Lande & Arnold, 1983). Such constraints can be investigated through quantitative genetics using the G‐matrix of additive genetic variances and covariances and the selection gradients, as has been done in a number of prior studies (Colautti & Barrett, 2011; Conner & Agrawal, 2005; Franks et al., 2012; Johnson et al., 2009; Smith & Rausher, 2008; Zu et al., 2020). Several different approaches have been used to quantify how much of a constraint would occur due to genetic correlations (Calsbeek & Goodnight, 2009), but a simple first approach is to compare direct and indirect selection, given that direct selection accounts for selection directly acting on a trait without accounting for correlations, while indirect selection includes selection driven by selection acting on other correlated traits (Conner & Hartl, 2004). If direct and indirect selection are similar, there is a lack of evidence that genetic correlations would constrain evolution. In our study, for almost all traits, direct and indirect selection measures were congruent. Thus, in general, genetic correlations would not be predicted to constrain evolution of drought response traits in this system.

The exception to this lack of constraint due to correlations was mass per seed (TGW) under drought, which showed negative direct selection but positive indirect selection. In this case, it appears that drought caused smaller seeds to be favored directly, but the plants would be expected to evolve larger seeds under drought due to correlations between seed size and other traits. This constraint and expected evolutionary outcome, as well as the trade‐off between seed mass and number, have important implications for crop breeding under drought, which is discussed below (Section 4.4).

### 4.3 Response to selection was also not constrained by antagonistic pleiotropy

With antagonistic pleiotropy, one gene causes different fitness consequences under different conditions (Kawecki & Ebert, 2004). The classic example is when a gene improves reproductive fitness early in life but reduces survival later in life. In this case, the gene may be favored and increase due to selection despite the negative consequences later in life, which have less of an effect on lifetime fitness. This is the antagonistic pleiotropy hypothesis for the evolution of senescence (Williams, 1957). With conditional neutrality, one gene is favored in one set of conditions but neutral in other conditions (Lascoux et al., 2016).

In our experimental populations of domesticated rice plants, we previously observed few instances of antagonistic pleiotropy at the level of gene expression (Groen et al., 2020). Here, we found that also at the functional trait level, there was a lack of evidence for antagonistic pleiotropy, given that GWAS did not detect individual genes that influenced more than one drought response trait and that patterns of selection were not opposite under different conditions. This demonstrates that the extent of pleiotropy in our populations was limited and that genetic correlations between the traits we studied were mostly driven by polygenic architectures. Thus, the presence of genetic correlations between traits was not explained by loci with large pleiotropic or linkage effects that we could detect. This finding contrasts with work showing that pleiotropy influenced genetic correlations for drought response strategies in *Arabidopsis thaliana* (McKay et al., 2003), which is possibly due to differences in the genetic architecture of drought response in these different species. It could also be due to differences in approach, with the earlier study using mutant and near‐isogenic lines, which could have facilitated detecting pleiotropy.

Our results further indicate that the few trade‐offs at the gene expression level found previously (Groen et al., 2020) most likely disappeared through buffering effects emerging at the post‐transcriptional level. Similar phenotypic buffering effects have been observed previously in *Arabidopsis thaliana* (Fu et al., 2009). Although our study only covered two different environments, the results provide further insight into the mechanism that reduces yield penalty on drought resistance in rice. Our results also add to growing evidence that conditional neutrality may be more common than antagonistic pleiotropy (Anderson et al., 2013; Crow et al., 2020; Fournier‐Level et al., 2011, 2013; Gardner & Latta, 2006; Leinonen et al., 2013; Lotterhos et al., 2018; Lowry et al., 2019; Lowry & Willis, 2010; Oakley et al., 2014; Price et al., 2018; Scarcelli et al., 2007; Soltani et al., 2017; Troth et al., 2018).

Although we did not find evidence for antagonistic pleiotropy, or pleiotropy more generally, based on GWAS, we did find that expression of several genes influenced more than one drought response trait, according to transcript–trait association analysis. The fact that we found a substantial set of overlapping transcripts between WUE, which is associated with drought avoidance, and DTF, which is associated with drought escape, supports a genetic basis for a trade‐off between these drought response strategies (Kenney et al., 2014; Kooyers, 2015). There were also notable cases in which we found genes with expression correlated to multiple traits that made sense in terms of the known function of these genes. For example, one transcript influenced both SSC and DTF, and the underlying gene is *DWARF18*/*OsGA3ox2* (Os01g0177400), which may be involved in regulating shoot elongation (Das et al., 2005; Itoh et al., 2001). Another gene, whose expression was linked to LRO, SSC, and WUE, was Os12g0291200, which encodes RuBisCo small subunit 3 (OsRBcS3) that forms an essential component of the photosynthetic machinery (Morita et al., 2014; Suzuki et al., 2007; Suzuki et al., 2009).

Despite the detection of some overlapping transcript–trait associations across traits, the results mostly point to a polygenic architecture for many traits, with limited evidence of pleiotropy driving trait correlations. Although expression variation of select sets of transcripts is associated with more than one trait, most traits appear to be regulated relatively independently by genetic and transcriptional variation of small effect in many loci. Based on our data, we suggest that pleiotropy across environments does not appear to be a major factor that would constrain the evolution of drought response traits in rice populations. Furthermore, this suggests that selection for crop improvement in one environment, such as one with water‐limited conditions, would not necessarily decrease yield in other environments, such as ones with more stable irrigation and water availability.

### 4.4 Seed size/seed number trade‐offs in crop and wild plants

A trade‐off between seed size and seed number represents a critical axis of variation in life history strategies within and between plant species, including in crops (Sadras, 2007), which is strongly influenced by water availability (Larios & Venable, 2018; Paul‐Victor & Turnbull, 2009; Smith & Fretwell, 1974). While we observed selection for smaller seeds in wet conditions, in line with a trade‐off between seed size and seed number, this trade‐off disappeared under drought. In particular, indirect selection (which was positive) opposed direct selection (which was negative) on seed mass (TGW). Selection on traits other than TGW thus changed the predicted outcome of selection on TGW under drought through genetic correlations.

What might these traits be? In the populations of both the Indica and Japonica panels, TGW showed relatively strong correlations with DTF, and early flowering was particularly intensely selected for under drought for both varietal groups. Interestingly, flowering time and seed size were found to be intimately linked in several mapping studies on wild populations of the plant *Arabidopsis thaliana* (Alonso‐Blanco et al., 1999; Gnan et al., 2014; Weinig et al., 2003). These genetic correlations were largely explained by pleiotropic loci of major effect, for example, *TERMINAL FLOWER1* (*TFL1*). This gene showed signatures of balancing selection linked to environmental heterogeneity (Weinig et al., 2003), and mutants in *TFL1* couple early flowering with large seed size (Zhang et al., 2020). However, in the case of the panels of rice accessions we studied, the genetic correlation between DTF and TGW does not appear to be caused by pleiotropic loci of large effect. This may be explained by the fact that during domestication, selective sweeps have occurred at many loci with large effects on seed size and flowering time (Paterson et al., 1995), such that the remaining genetic correlations are probably mostly polygenic in nature.

Decades of breeding for drought tolerance at IRRI have yielded several important insights with regard to traits related to drought resistance, and our results may provide partial mechanistic explanations to accompany these. First, major‐effect QTL related to drought resistance traits are typically only observed under drought and not in wet conditions (Kumar et al., 2014, 2021). Our observation in Indica of higher heritability for several physiological and life history traits in dry than in wet conditions, combined with the observation that traits typically show conditional neutrality when under selection rather than antagonistic pleiotropy, suggests that there may indeed be little variation for some traits unless plants encounter drought, in which case they then show variation for the response. Second, beneficial values for trait combinations, rather than single traits alone, are observed in breeding lines with the best yield under drought (Kumar et al., 2014, 2021). In our study, we found that although yield in wet conditions is constrained by the seed size/seed mass trade‐off, genetic correlations between a combination of traits appeared to alleviate negative selection for smaller seeds under drought so that yield could continue to improve as fitness improved. This finding further emphasizes that it is important to include estimates of the predicted outcome of selection when performing selection analysis, as genetic correlations can be critical for predicting evolutionary changes in suites of traits (Svensson et al., 2021).

Our results on the strength and patterns of natural selection on drought resistance traits in rice may provide a partial a posteriori explanation for why it was possible for rice breeders to develop drought resistance breeding lines such as the widely released variety Sahbhagi Dhan without a yield penalty in wet conditions (Anantha et al., 2016; Kumar et al., 2021). We show that plausible reasons for a lack of constraint on responses to drought selection were a lack of genetic correlations opposing selection under different conditions as well as a lack of antagonistic pleiotropy. Performing field experiments and selection analyses like ours with a diverse sampling of available germplasm prior to setting up new breeding programs could help with providing the confidence and justification for embarking on years of breeding trials to develop novel stress‐tolerant crop varieties. Finally, the landraces, candidate genes, and genetic variants we identified as being associated with drought resistance represent valuable resources for future functional characterization toward enhancement of stress‐resistant rice breeding lines.

## CONFLICT OF INTERESTS

The authors declare no conflict of interest.

## Supporting information


Figure S1
Figure S2
Figure S3
Figure S4
Figure S5
Figure S6

Appendix S1

Table S1

Table S2

Table S3

Table S4

Table S5

Table S6

## Data Availability

Data from this study are archived in the NCBI Short Read Archive (ID number PRJNA557122) and Zenodo (DOI: 10.5281/zenodo.5513036).
