# Supplementary figures and images for: The influence of genetic architecture on responses to selection under drought in rice
Source: Evol Appl. 2022 Jun 6;15(10):1670–90. doi: 10.1111/eva.13419 (PMC9624088; doi:10.1111/eva.13419)

1

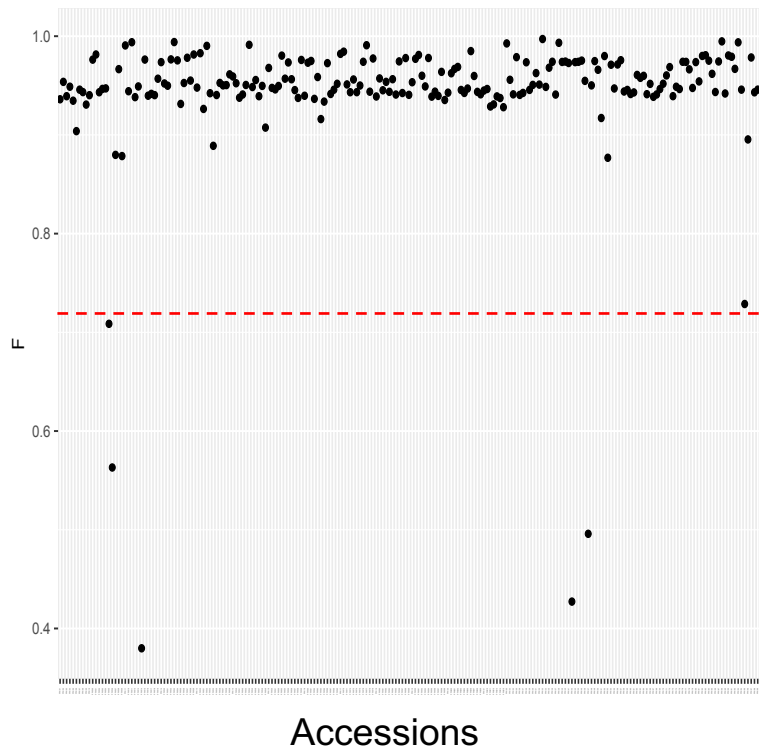

2

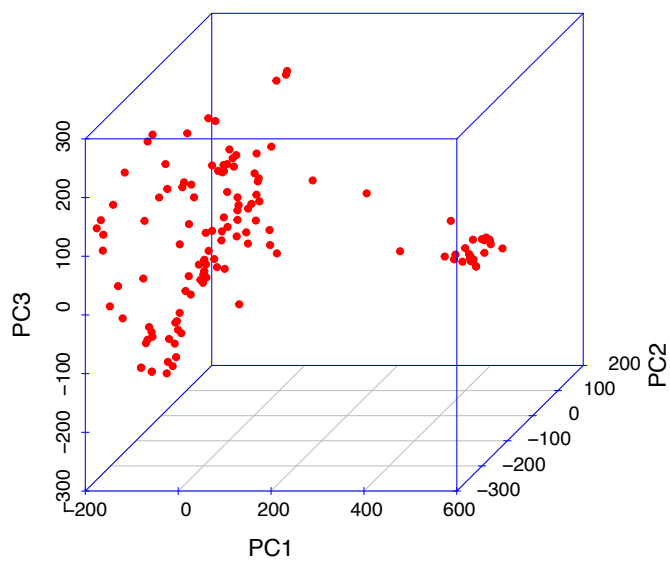

3

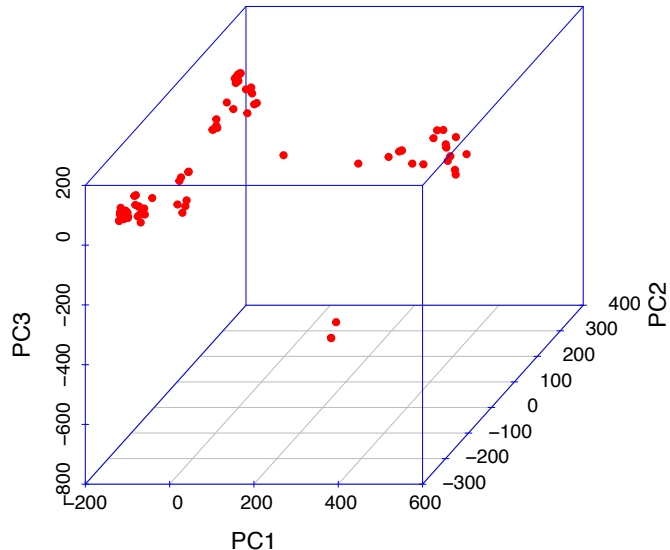

4

Wet

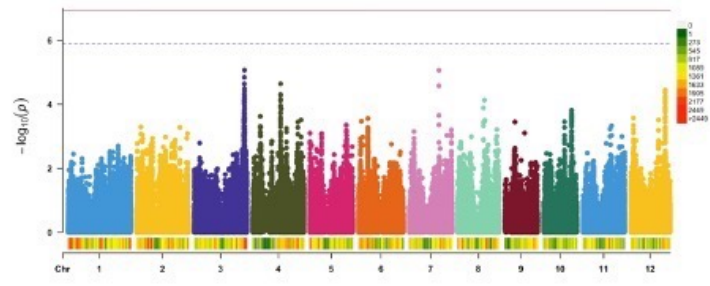

LOP

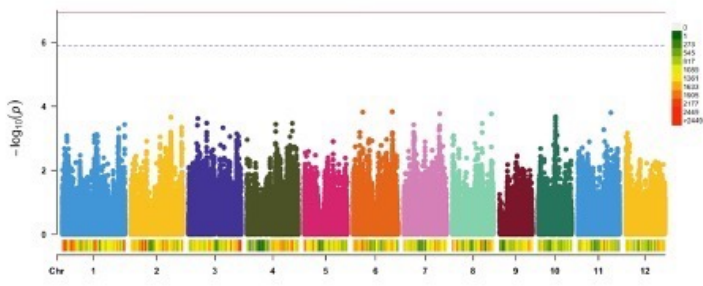

Dry

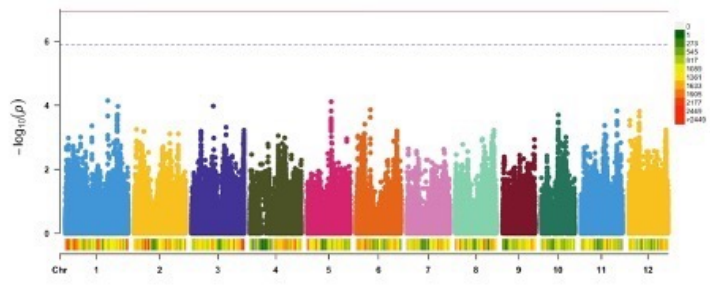

WUE

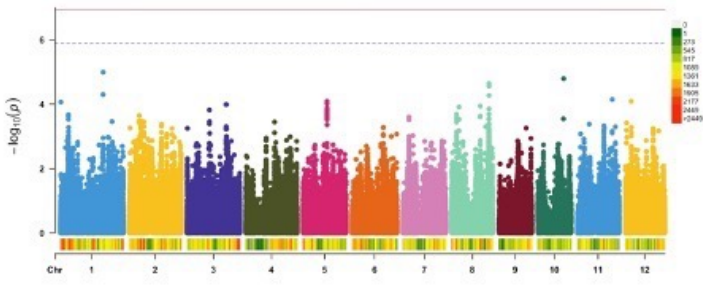

XHS

INDICA

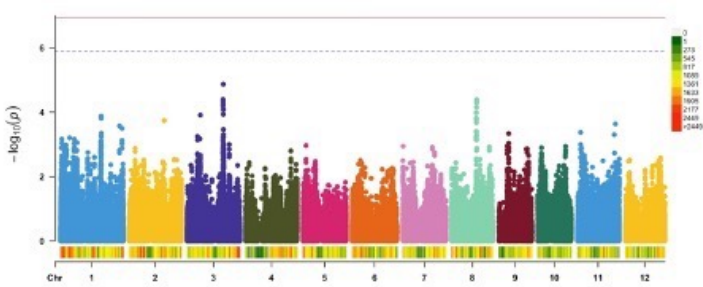

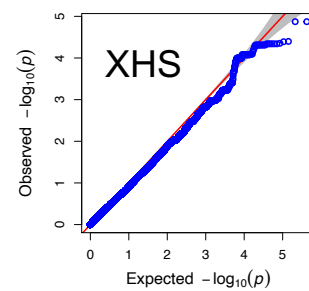

6

Wet

LOP

Dry

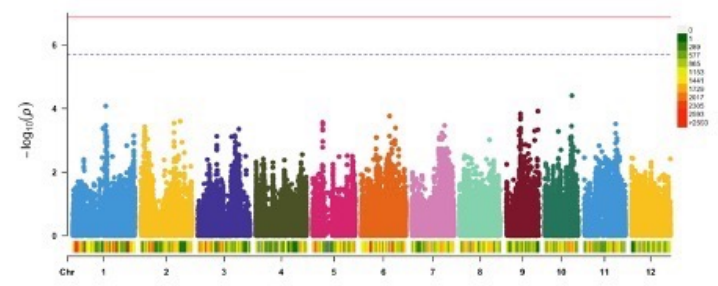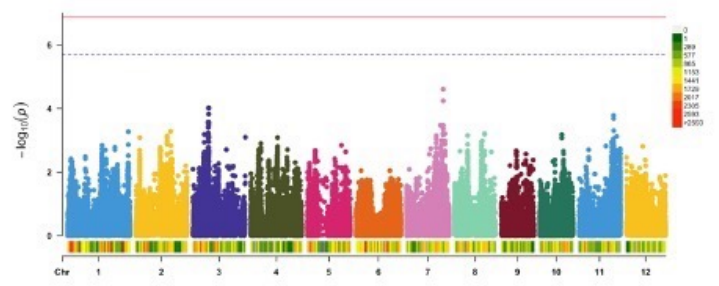

WUE

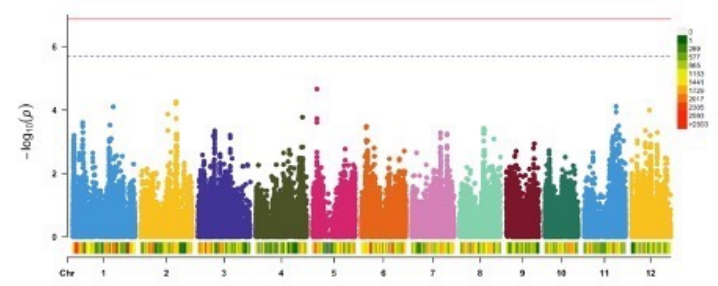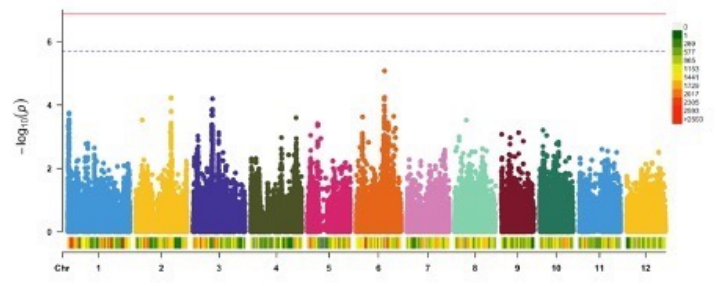

XHS

JAPONICA

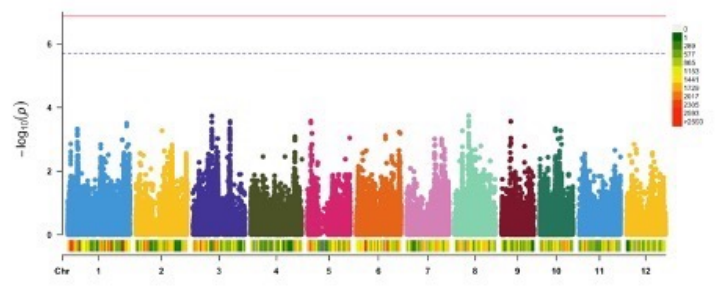

Wet

Dry

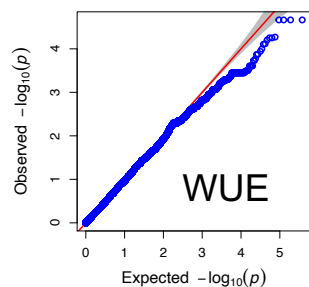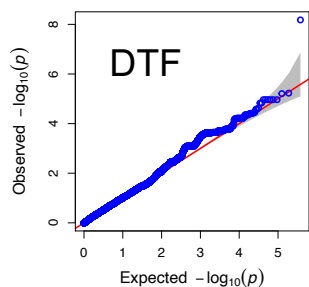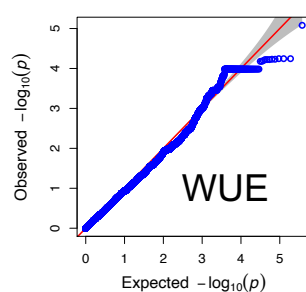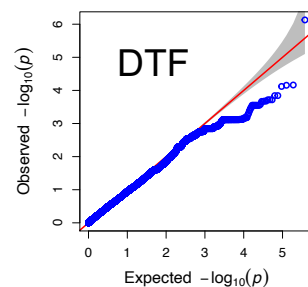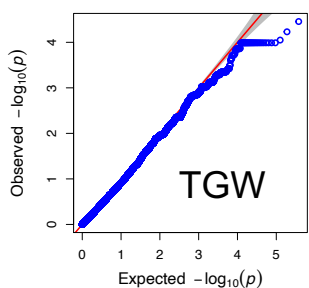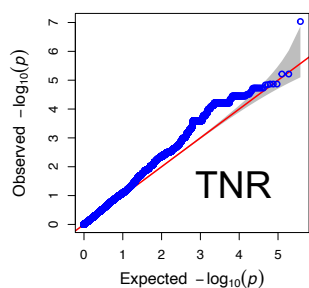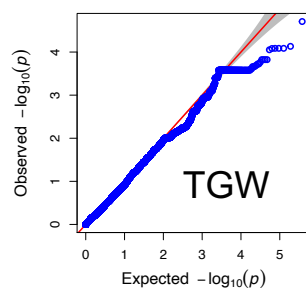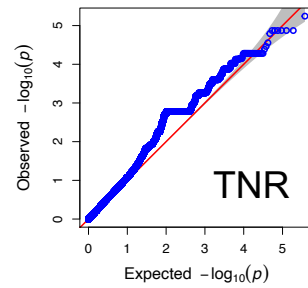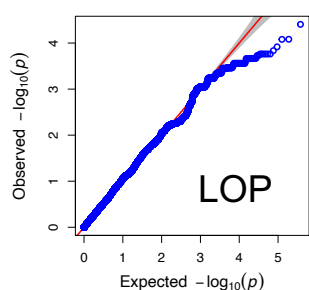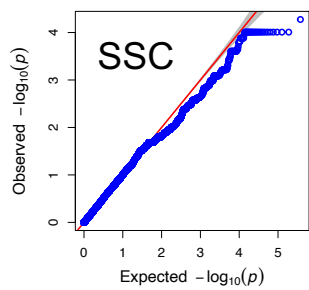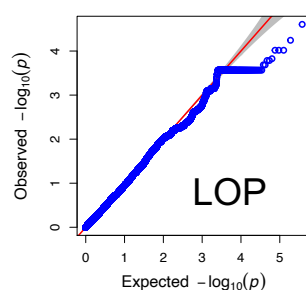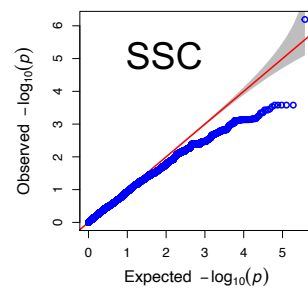

JAPONICA

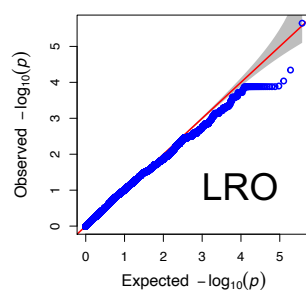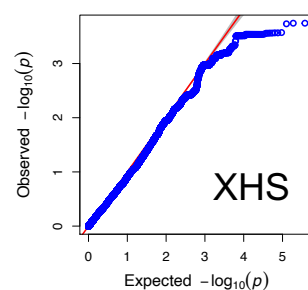

8

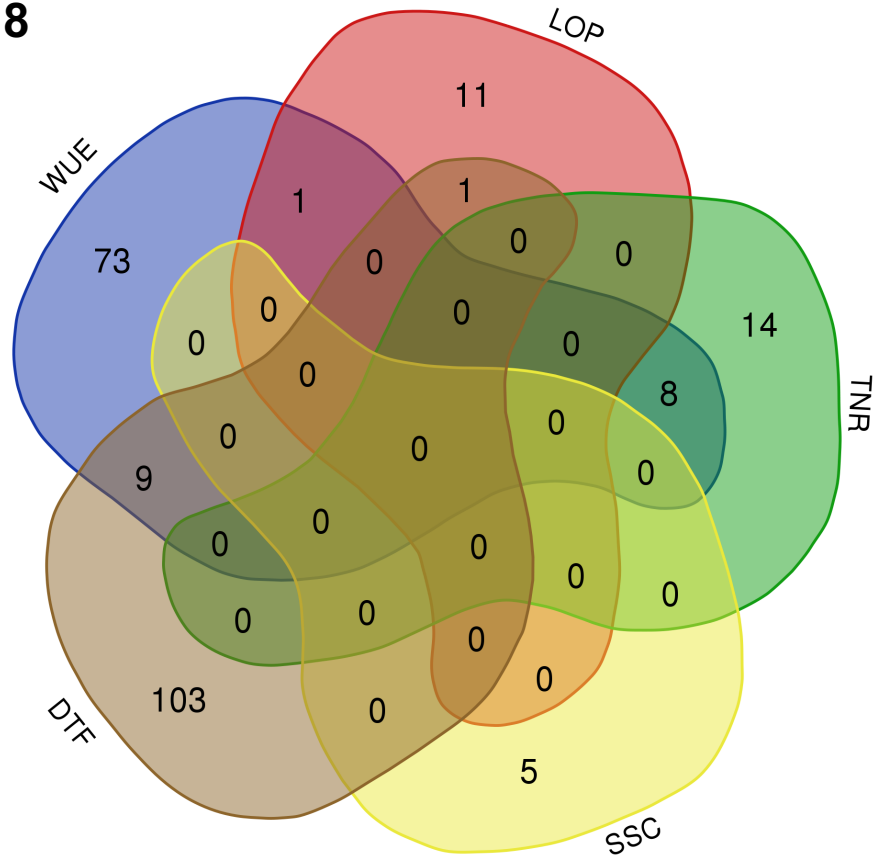

Supplement: Supplementary file 1 — Figure S1 Figure S2 Figure S3 Figure S4 Figure S5 Figure S6 [file EVA-15-1670-s007.pdf]
