# Supplementary material for: The influence of genetic architecture on responses to selection under drought in rice
Source: Evol Appl. 2022 Jun 6;15(10):1670–90. doi: 10.1111/eva.13419 (PMC9624088; doi:10.1111/eva.13419)
Supplement: Supplementary file 2 — Appendix S1 [file EVA-15-1670-s001.docx]

**Supporting Information**

**Supplementary Note 1**

A script for phenotypic selection analysis using a Linear Regression Mixed Model with block effect can be found here: https://github.com/icalic/Linear-Regression-Mixed-Model-with-block-effect.git

**Supplementary Note 2**

A script for phenotypic selection analysis using a Logistic Regression Mixed Model with block effect can be found here: https://github.com/icalic/Logistic-Regression-Mixed-Model-with-block-effect.git

**Supplementary Note 3**

We used the following ANGSD command for obtaining genotype posterior probabilities: angsd -b $BAM_list -ref $Reference_Genome -anc $Outgroup_genome \ -out $GPP \ -uniqueOnly 1 -remove_bads 1 -only_proper_pairs 1 -trim 0 -C 50 -baq 1 \ -minMapQ 30 -minQ 30 \ -minInd $minInd -setMinDepth $setMinDepth -setMaxDepth $setMaxDepth \ doCounts 1 -GL 1 -doMajorMinor 1 -doMaf 1 –skipTriallelic 1 \ -SNP_pval 1e-3 -doGeno 32 -doPost 1

**Supplementary Note 4**

A script for the calculation of LS means can be found here: https://github.com/icalic/Least-square-means-LSmeans-.git

**Supplementary Note 5**

A script for the calculation of SimpleM *P* value thresholds can be found here: http://sourceforge.net/projects/simplem/files/simpleM_Ex.zip/download

**Supplementary Note 6**

Files related to the estimation of SNP-based heritability, GWAS and genomic prediction analysis have been deposited in Zenodo (DOI: 10.5281/zenodo.5513036). For GWAS and genomic prediction analysis, we used the GAPIT software package (Lipka et al., 2012). GAPIT calculates a kinship matrix for a population under study using the VanRaden method (VanRaden, 2008), conducts GWAS, and performs genomic prediction at the optimum compression level using the default clustering algorithm (Average) and group kinship type (Mean). In our analysis, we specified the “PCA. Total” parameter as equal to 3 to specify the number of principal components (PCs) to include in the GWAS model. Our GWAS model was a multi-locus mixed model (Segura et al., 2012). MLMM uses forward-backward stepwise linear mixed-model regression, including associated markers as covariates.

**Supplementary Note 7**

Descriptions of candidate genes near SNPs associated with traits in populations of our rice diversity panels can be found below:

Indica Wet Environment

For TGW, the top SNP chr02:7,617,700 was located 17,832 bp downstream of Os02g0234200 (chr02:7,594,952..7,599,868; +strand). This gene codes for an NHL domain-containing protein named FUWA, and a previous examination of a diverse set of rice landrace and wild relative accessions suggested that regulatory variation in this gene might cause variation in grain weight and other traits (Chen et al., 2015). For TNR, the top SNP chr01:15,017,932 was located 19,829 bp upstream of Os01g0367100 (chr01:14,993,204..14,998,103; -strand). This gene, *PHD1*, was introgressed into Indica from Japonica (Seo et al., 2020), and allelic variation governs phenotypic differences in photosynthetic efficiency, tillering, and grain yield between accessions (Abbai et al., 2019; Li et al., 2011). For SSC, the top SNP chr06:1645106 was located 11,599 bp downstream of Os06g0130400 (chr06:1,629,778..1,633,507; +strand). The gene codes for ACC SYNTHASE 6 (OsACS6) / SUBSTANDARD STARCH GRAIN 6 (OsSSG6) and it influences starch metabolism via its role in ethylene signaling (Matsushima et al., 2016). Interestingly, allelic variation was also introgressed into Indica from Japonica (Seo et al., 2020).

Indica Dry Environment

For TGW, the top SNP chr07:21,846,044 had three drought-responsive S-domain subfamily of receptor-like kinases (SDRLKs) located within 50kb up- and downstream of it (Naithani, Dikeman, Garg, Al-Bader, & Jaiswal, 2021). For LRO, the top SNP chr09:15,043,804 was located 32,454 bp upstream of Os09g0419200 (chr09:15,076,258..15,079,326; -strand). This gene codes for CINNAMOYL-COA REDUCTASE 19 (OsCCR19), and regulates developmental lignification (Park, Bhoo, Kwon, Lee, & Cho, 2017), which in turn may influence leaf rolling (Li et al., 2017; Zhang, Xu, Zhu, Qian, & Xue, 2009).

Japonica Wet Environment

For TNR, the top SNP chr03:22,820,285 was located 24,641 bp upstream of Os03g0607600 (chr03:22,844,926..22,847,390; + strand). This gene codes for Cyclin-A3-1; cyclins have been linked to tillering in several mutant studies (Corvalán, An, & Choe, 2021; Xu et al., 2012). For DTF, the top SNP chr03:1,292,315 had two candidate loci located within 50kb up- and downstream of it: Os03g0122500 gives rise to the long non-coding RNA *Ef-cd* (*Early flowering-completely dominant*) that stimulates early flowering (Fang et al., 2019), while *OsMADS50* has a well-established role as a flowering regulator (Bian et al., 2011).

Japonica Dry Environment

For DTF, the top SNP chr06:10,914,098 appears to tag an expanse of transposable elements downstream of Os06g0298200 (chr06:11,070,174..11,076,691; -strand). This gene codes for OsCCT22, a known regulator of flowering time (Zhang et al., 2021). For SSC, the top SNP chr06:21,502,702 was located 20,321 bp downstream of Os06g0561000 (chr06:21,478,477..21,482,381; +strand). This gene encodes OsMIOX, differential expression of which impacts transcript levels of genes involved in sugar metabolism, ROS scavenging and hormone signaling, impacting drought resistance (Duan et al., 2012; Shi, Dong, Wang, & Qiu, 2020).

**Supplementary Note 8**

Descriptions of transcripts of interest associated with traits in populations of our rice diversity panels can be found here:

Indica Wet Environment

SSC had expression of OS04T0624600-02, which encodes SOLUBLE STARCH SYNTHASE IIIB, associated with it. The molecular function of this protein fits with determining sugar content (Table S4). In addition, we found further evidence for a previously hypothesized influence on WUE of the S-like ribonuclease OsRNS4 (encoded by OS09T0537700-02; Zheng et al., 2014), and phytochrome signaling, here through OsPIF3 (encoded by OS01T0286100-01; Gao et al., 2015).

Japonica Wet Environment

Flowering-related GO terms were further enriched among transcripts associated with TNR, while transcripts linked to LOP were enriched for ion transporters (Table S4).

Indica Dry Environment

Among transcripts associated with DTF in Indica was OS03T0122500-01, which gives rise to the long non-coding RNA *Ef-cd* (*Early flowering-completely dominant*). The gene encoding *Ef-cd* was also among the candidate genes near a SNP that was associated with flowering time in Japonica grown in wet conditions.

Japonica Dry Environment

Transcripts associated with SSC were strongly enriched for ones involved in photosynthesis (*P* = 3.7×10^-11^), just as we found to be the case for transcripts associated with both SSC and WUE.

**Supporting Information References**

Abbai, R., Singh, V. K., Nachimuthu, V. V., Sinha, P., Selvaraj, R., Vipparla, A. K., … Kumar, A., 2019. Haplotype analysis of key genes governing grain yield and quality traits across 3K RG panel reveals scope for the development of tailor‐made rice with enhanced genetic gains. *Plant Biotechnology Journal*, 17(8), 1612-1622.

Bian, X. F., Liu, X., Zhao, Z. G., Jiang, L., Gao, H., Zhang, Y. H., … Wan, J. M. (2011). Heading date gene *Dth3* controlled late flowering in *O. glaberrima* Steud. by down-regulating *Ehd1*. *Plant Cell Reports*, 30(12), 2243-2254.

Chen, J., Gao, H., Zheng, X. M., Jin, M., Weng, J. F., Ma, J., … Wang, J. L. (2015). An evolutionarily conserved gene, *FUWA*, plays a role in determining panicle architecture, grain shape and grain weight in rice. *Plant Journal*, 83(3), 427-438.

Corvalán, C., An, G., & Choe, S. (2021). The Rice *propiconazole resistant 1-D* mutant, with activated expression of a DPb transcription factor gene, exhibits increased seed yields. *bioRxiv*, 425087.

Duan, J., Zhang, M., Zhang, H., Xiong, H., Liu, P., Ali, J., … Li, Z. (2012). *OsMIOX*, a myo-inositol oxygenase gene, improves drought tolerance through scavenging of reactive oxygen species in rice (*Oryza sativa* L.). *Plant Science*, 196, 143-151.

Fang, J., Zhang, F., Wang, H., Wang, W., Zhao, F., Li, Z., … Chu, C. (2019). *Ef-cd* locus shortens rice maturity duration without yield penalty. *Proceedings of the National Academy of Sciences of the United States of America*, 116(37), 18717-18722.

Gao, Y., Jiang, W., Dai, Y., Xiao, N., Zhang, C., Li, H., … Chen, J. (2015). A maize phytochrome-interacting factor 3 improves drought and salt stress tolerance in rice. *Plant Molecular Biology*, 87(4-5), 413-428.

Li, C., Wang, Y., Liu, L., Hu, Y., Zhang, F., Mergen, S., … Chu, C. (2011). A rice plastidial nucleotide sugar epimerase is involved in galactolipid biosynthesis and improves photosynthetic efficiency. *PLoS Genetics*, 7(7), e1002196.

Li, W. Q., Zhang, M. J., Gan, P. F., Qiao, L., Yang, S. Q., Miao, H., … Shi, C. H. (2017). CLD 1/SRL 1 modulates leaf rolling by affecting cell wall formation, epidermis integrity and water homeostasis in rice. *Plant Journal*, 92(5), 904-923.

Lipka, A. E., Tian, F., Wang, Q., Peiffer, J., Li, M., Bradbury, P. J., … Zhang, Z. (2012). GAPIT: genome association and prediction integrated tool. *Bioinformatics*, 28(18), 2397-2399.

Matsushima, R., Maekawa, M., Kusano, M., Tomita, K., Kondo, H., Nishimura, H., … Sakamoto, W. (2016). Amyloplast membrane protein SUBSTANDARD STARCH GRAIN6 controls starch grain size in rice endosperm. *Plant Physiology*, 170(3), 1445-1459.

Naithani, S., Dikeman, D., Garg, P., Al-Bader, N., Jaiswal, P. (2021). Beyond gene ontology (GO): using biocuration approach to improve the gene nomenclature and functional annotation of rice S-domain kinase subfamily. *PeerJ*, 9, e11052.

Park, H. L., Bhoo, S. H., Kwon, M., Lee, S. W., Cho, M. H. (2017). Biochemical and expression analyses of the rice *cinnamoyl-CoA reductase* gene family. *Frontiers in Plant Science*, 8, 2099.

Segura, V. *et al*. (2012). An efficient multi-locus mixed-model approach for genome-wide association studies in structured populations. *Nature Genetics* 44, 825-830.

Seo, J., Lee, S. M., Han, J. H., Shin, N. H., Lee, Y. K., Kim, B., … Koh, H. J. (2020). Characterization of the common Japonica-originated genomic regions in the high-yielding varieties developed from inter-subspecific crosses in temperate rice (*Oryza sativa* L.). *Genes*, 11(5), 562.

Shi, F., Dong, Y., Wang, M., & Qiu, D. (2020). Transcriptomics analyses reveal that OsMIOX improves rice drought tolerance by regulating the expression of plant hormone and sugar related genes. *Plant Biotechnology Reports*, 14(3), 339-349.

VanRaden, P.M. (2008). Efficient methods to compute genomic predictions, *J. Dairy Sci* 91, 4414-4423.

Xu, C., Wang, Y., Yu, Y., Duan, J., Liao, Z., Xiong, G., … Li, J. (2012). Degradation of MONOCULM 1 by APC/C TAD1 regulates rice tillering. *Nature Communications*, 3(1), 750.

Zhang, G. H., Xu, Q., Zhu, X. D., Qian, Q., & Xue, H. W. (2009). SHALLOT-LIKE1 is a KANADI transcription factor that modulates rice leaf rolling by regulating leaf abaxial cell development. *Plant Cell*, 21(3), 719-735.

Zhang, J., Fan, X., Hu, Y., Zhou, X., He, Q., Liang, L., & Xing, Y. (2021). Global analysis of *CCT* family knockout mutants identifies four genes involved in regulating heading date in rice. *Journal of Integrative Plant Biology*, 63(5), 913-923.

Zheng, J., Wang, Y., He, Y., Zhou, J., Li, Y., Liu, Q., & Xie, X. (2014). Overexpression of an S-like ribonuclease gene, *OsRNS4*, confers enhanced tolerance to high salinity and hyposensitivity to phytochrome-mediated light signals in rice. *Plant Science*, 214, 99-105.
